# Compaction Curves and Strength of Clayey Soil Modified with Micro and Nano Silica

**DOI:** 10.3390/ma15207148

**Published:** 2022-10-14

**Authors:** Abeer W. Alshami, Bashar H. Ismael, Mohammed F. Aswad, Ali Majdi, Murtatha Alshijlawi, Mustafa Mohammed Aljumaily, Mohamed Khalid AlOmar, Ibraheem A. Aidan, Mohammed Majeed Hameed

**Affiliations:** 1 Civil Engineering Department, Al-Maarif University College (AUC), Ramadi 31001, Iraq; 2 Civil Engineering Department, University of Technology, Baghdad 10066, Iraq; 3 Department of Building and Construction Technics Engineering, Al Mustaqbal University College, Hilla 51001, Iraq; 4 Technical Institute of Anbar, Middle Technical University, Falluja 31002, Iraq; 5 Department of Civil Engineering, Faculty of Engineering and Built Environment, Universiti Kebangsaan Malaysia, Bangi 43600, Selangor, Malaysia

**Keywords:** clayey soil, soil improvement, silica fume, nanomaterial

## Abstract

Some Clayey soils are generally categorized as weak soils, and structures lying on such soils have been exposed to severe damage. Therefore, the central thesis of this paper is the impact of a waste material known as a silica fume as nano and micro material on soil’s behaviour. To evaluate the effects of those additives on Atterberg limits, compaction characteristics and unconfined compressive strength, clayey soil samples have been transformed using micro and nano silica fume (by-product materials). In the current investigation, silica fume is used at four different percentages: 0, 2, 4, and 7%. The results show that the plasticity index of soil decreases with the addition of micro silica and increases with the addition of nano-silica. Increasing nano silica percentage improves the dry density of the compacted soil and reduces the optimum moisture content. An opposite behavior is observed with adding micro silica to compacted soil. Finally, 4% of silica fume is found to be the optimum dosage to improve the unconfined compressive strength of the treated soil with both additives. As a result, treating the weak clay soil with micro and/or nano-silica fume has the potential to be impactful.

## 1. Introduction

One of the most prevalent issues worldwide is the location of civil engineering projects in areas with unstable soils. Weak soil can cause severe damage to buildings and infrastructures, especially when the ground is liquefiable [1,2,3,4,5,6,7,8,9]. The traditional approach to stabilizing soil involves removing the brittle soil and replacing it with a more potent substance. The high expense of this technology has prompted academics to hunt for cheaper alternatives, one of which is the soil stabilization procedure.

A technique called soil stabilization was first used to make soils capable of satisfying the demands of particular engineering projects many years ago [10]. Soils may need to be stabilized if they are poor or have unwanted characteristics that make them inappropriate for use in a geotechnical project. Several scientific methods for stabilizing soil have been developed recently. Several studies have been conducted to improve the soil using additives such as fly ash, cement, and lime. The techniques of soil stabilization frequently involve additives as cementing agents, including cement, lime, or industrial by-products [11]. Historically, lime, cement, and specialized additives like pozzolanic materials have been used to treat soils that make up the pavement subgrade to stabilize them. Fly ash, silica fume, and rice husk ash, considered wastes, are pozzolanic materials that can be utilized to enhance soil [12].

Due to its tiny particles, large surface area, and high silicon dioxide concentration, silica fume, one of the stabilizers, has drawn the most interest as a highly reactive pozzolan [13]. Portland cement was partially substituted with silica fume in concrete. Additionally, silica fume has been suggested as a promising and effective alternative to enhance the geotechnical characteristics of clayey soils as a stabilizing agent by enhancing unconfined compressive strength and decreasing the permeability coefficient [14]. This stabilizer can improve composite permeability, swelling pressure, and compressive strength, according to research on the impact of silica fume on the geotechnical parameters of high plasticity clay [15]. Additionally, pozzolanic additions were employed to enhance the properties of swelling soils [16]. After the curing process, these additives can significantly reduce the dispersivity potential and plasticity index while increasing the soils’ unconfined compressive strength (UCS). To improve the material properties used in many engineering applications, particularly in civil engineering, nanomaterials and nanoparticles are extensively used as additives nowadays [17]. Nanomaterials have many advantages when used as stabilizers. A material’s relative surface area can impact materials’ strength or electrical qualities, making them more chemically reactive.

Additionally, quantum effects can cause materials’ optical, electrical, and magnetic properties to dominate their behavior at the nanoscale. The most significant benefits of nanomaterials to improve quality of life and healthier lifestyles have been outlined as reducing energy usage, saving money, saving time, and improving the quality of products. Nanomaterials are thought to be a potentially strong stabilizer to enhance the characteristics of soils based on the previously mentioned advantages [18].

Due to its low strength traits, clay is regarded as soil with weak properties. Based on the justifications mentioned above, several studies used silica fume and nano-silica as regularly used additives to enhance the geotechnical characteristics of various clay soils [17]. For the reasons already stated, a study is being done to determine the influence of a waste material known as-silica-fume as nano and micro material on poor clay, also known as Hujaira’s clay, which is now being used as a filler material beneath projects being implemented in Fallujah, Iraq, due to the lack of suitable soil.

The tests adopted in this research namely Atterberg limits, compaction, and unconfined compressive strength (UCS) tests on stabilized soil, were conducted to achieve three goals: at first, to improve the characteristics of such problematic soils so that they can be used confidently in engineering practice; secondly to determine the ideal dosage needed to improve the soil, and lastly to enhance the environmental impact by utilizing the use of a by-product material called silica fume.

## 2. Materials and Methods

### 2.1. Clayey Soil

**Figure 1:**
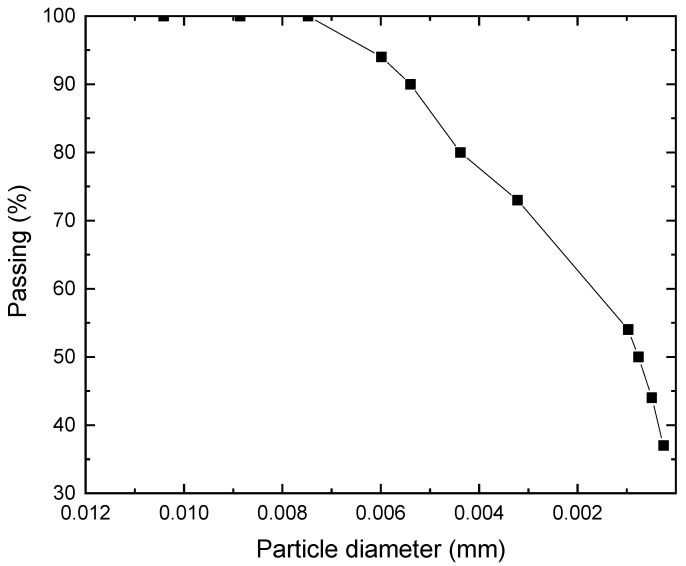
Grain size distribution of soils.

A soil sample from Al-Fallujah City in Iraq was brought over for this study. This soil was taken as undisturbed sample using 100 mm tube with less than 20% area ratio (i.e., undisturbed sample) at a depth ranging from 0.5 to 1 m from the borrow pits. All soil samples are packaged in plastic bags, identified, and sent to the Al Maaref University College Soil Mechanics Laboratory for testing (AUC). To obtain this soil to the soil mechanics lab, plastic bags were used to carry it. Figure 1 displays the soil’s distribution of particle sizes. Several tests, including sieve analysis, specific gravity (Gs), Atterberg’s limits (LL and PL), compaction testing, and unconfined compression testing, are carried out in accordance with established requirements to address the geotechnical properties of the soils employed. The physical characteristics of the clay soil used in this study are shown in Table 1.

### 2.2. Silica-Fume

Historically, silica fume, a very fine solid particle produced during silicon metal manufacturing, has been considered a waste. It is a secondary product of ferrosilicon alloys or silicon metal manufacture. Despite being an industrial waste, silica fume now ranks among the most effective secondary product pozzolanic materials because of its highly active pozzolanic properties. Concrete is one of silica fumes’ best applications. Due to its chemistry and physics, it is a highly reactive pozzolan [15,19,20,21]. However, Table 2 provides an overview of its chemical and index characteristics.

A pozzolanic substance with a high concentration of amorphous silicon dioxide and very small, spherical particles is known as a grey-colored, densified silica fume. Calcium silicate hydrate is created as a result of its reaction with calcium hydroxide (secondary gel).

The surface area of the very small vitreous particles that make up silica fume weighs 20,000 m^2^/kg. These particles are smaller by two orders of magnitude than the cement particle’s average size. Due to its extreme fineness and high silica concentration, silica fume is classified as a reactive pozzolanic material [15].

Additionally, it meets the ASTM C618 pozzolana chemical standard, as shown in Table 3.

### 2.3. Preparation of Nano-Materials

Depending on their use, nanometers are defined in many ways. Typically, in the nano range, particles between the sizes of 1 nm and 100 nm are referred to as ultrafine particles, while those between the sizes of 100 nm and 250 nm are considered finer particles. The materials are, therefore, in the finer nanometer particle range, according to the results of the particle size study [22].

The following process was utilized to convert the macro materials into nanomaterials, which were then used as additions to strengthen the weak soil investigated in this study:

First, the stabilizing substance, silica fume, must be oven-dried. The sample is either pulverized for 10–14 days or 10,000 revolutions in a ball mill.

**Figure 2:**
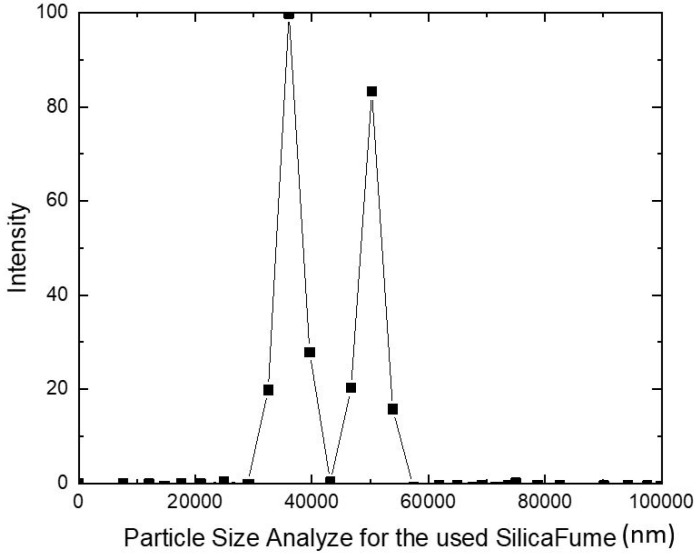
Intensity-particle size for the used additive-silica fume.

The continual pulverization of the material sample is a challenging process since the material particles adhere to the cylinder wall. For consistent pulverization, the cylinder should be cleaned every four hours. As demonstrated in Figure 2, the Particle Size Analyser analyses the fine ground sample to determine the particle size.

The Dynamic Light Scattering Principle underlies how the Particle Size Analyser operates (DLS). Measurements of particle size range from 0.3 nm to 8 m. Dispersing agents like sodium hexametaphosphate, sodium carbonate, or KNO_3_ should be used to spread the sample. As a result, 1 mg of the sample is dissolved in ethanol and placed in a test tube for 30 min to distribute the material particles evenly. The test tube is then kept inside the device for examination. Nano silica fume has particle sizes between (100–1000) nm and an effective diameter of 408.35 nm, while nano fly ash has particle sizes between (10–1000) nm and an effective diameter of 808.22 nm. The nanomaterial employed in this study is thought to be coarser nanoscale particles with a three-dimensional nanostructure.

### 2.4. Test Methods

As mentioned before, the central aim of this research is to investigate the impact of a waste material known as silica fume as nano and micro material on soil’s behavior of the clayey soil that was employed in the research. The following tests were run to further the research’s objectives:

#### 2.4.1. Atterberg Tests (Consistency Limits)

Atterberg limits were assessed to ascertain the impact of silica fume on the consistency behavior of compacted clayey soil samples. According to ASTM D 4318, the natural and stabilized clayey soil samples were put through liquid and plastic limits.

#### 2.4.2. Compaction Tests

In line with ASTM D 698, Standard Proctor tests were performed on soil samples of natural and stabilized clayey soil to determine the ideal water concentrations. The values of the ideal water content and maximum dry unit weight were calculated from the compaction curves shown. To produce samples for the unconfined compressive strength, the native clayey soil and the clayey soil-silica fume or nano-silica combinations were compacted at the ideal water content.

#### 2.4.3. Unconfined Compressive Strength Tests

Unconfined compression tests were used to measure the compressive strength of samples of compacted clay with silica fume and nano silica (ASTM 2166). The unconfined compression test is a popular and quick way to determine the approximate compressive strength of cohesive soils. Four groups of samples with a length/diameter ratio of 2 were prepared for this laboratory experiment (L: 70 mm and D: 35 mm).

### 2.5. Results and Discussion

#### 2.5.1. Atterberg Limits

**Figure 3:**
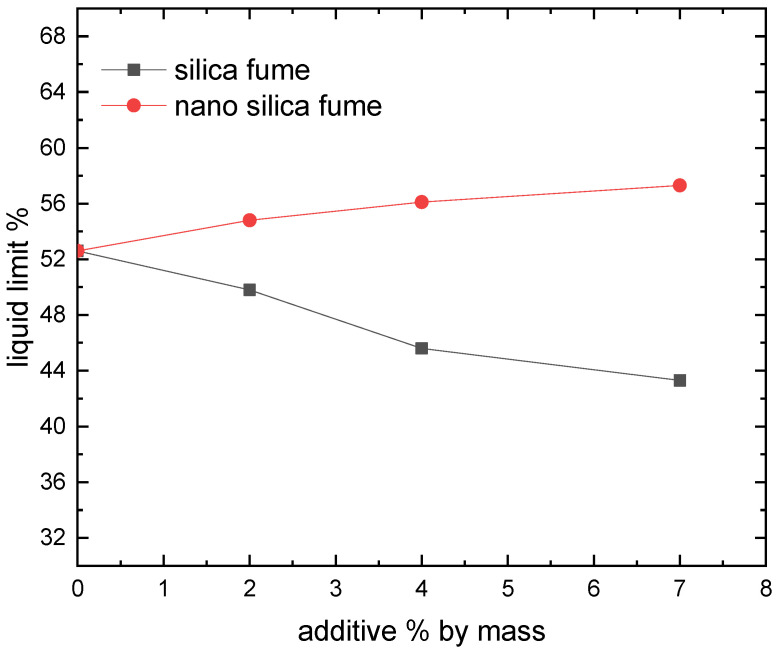
Effect of silica fume and nano silica on liquid limit.

**Figure 4:**
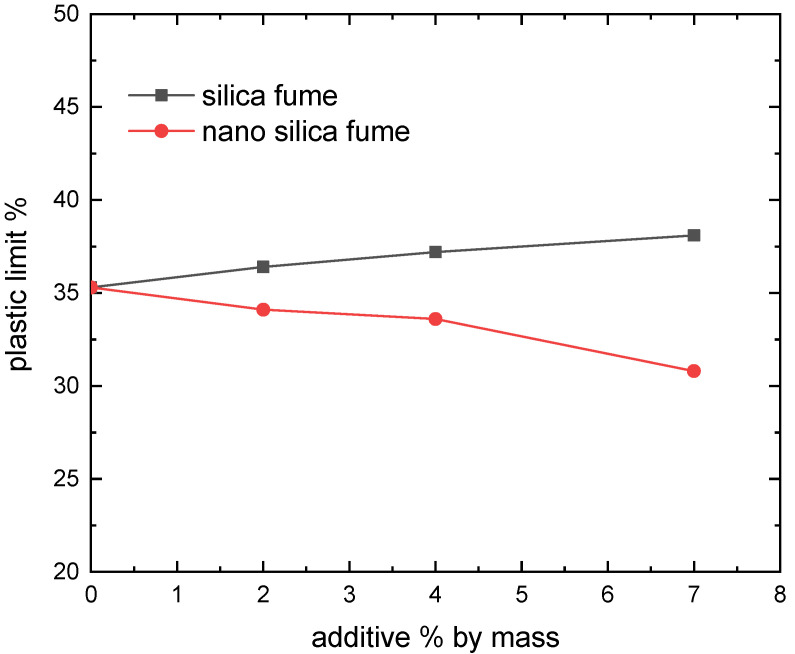
Effect of silica fume on Plastic limit.

**Figure 5:**
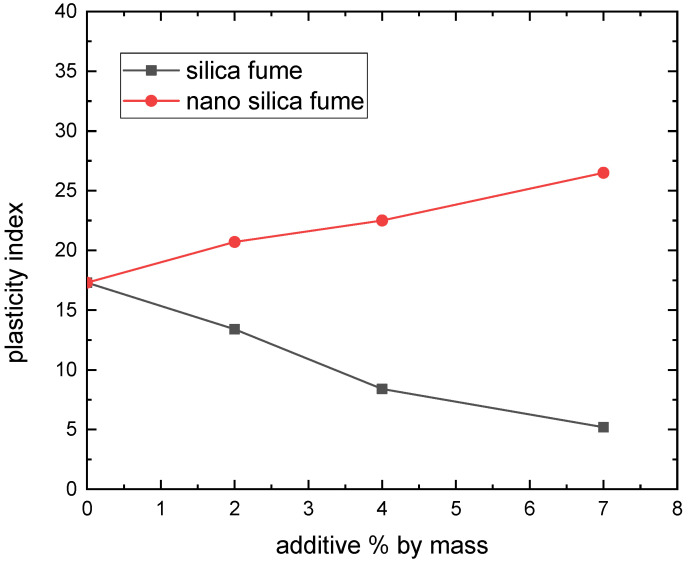
Effect of silica fume on plasticity index.

The Atterberg limits test assessed how silica fume and nano-silica affected the soil’s plastic properties. The mean liquid limit (LL), plastic limit (PL), and plasticity index (PI = LL-PL) of three replicates for each specimen are displayed in Figure 3, Figure 4 and Figure 5. With the addition of silica fume, the liquid limit and plasticity index were reduced. This might be influenced by the kind of soil and its cation exchange capability [14]. These findings were consistent with earlier research [14,23]. In thiss context, Kalkan and Akbulut showed that higher silica fume content of clay soil by up to 50% causes the liquid limit and plasticity index to drop [14]. As the silica fume level rises, it has also been demonstrated that the plasticity index of smectite clay somewhat declines [23]. Silica fume coats and binds all clay particles, even those with limited cementitious value and big particles, in a process known as the pozzolanic reaction between silica fume and aluminous material, which results in a decrease in the LL and PI [24]. The plastic limit reduced as the nano-silica content rose, according to the testing findings depicted in Figure 3, Figure 4 and Figure 5. The liquid limit and plasticity index increased. When the dose of nano-silica exceeds 1%, it can be attributed to the agglomeration of nanoparticles [25]. Due to their petite size, nano silica particles have a high specific surface. The amount of water adsorbed and the wettable surface area would rise with a high specific surface material [26]. These characteristics may raise the plasticity index and liquid limit while decreasing the plastic limit.

#### 2.5.2. Compaction Parameters

**Figure 6:**
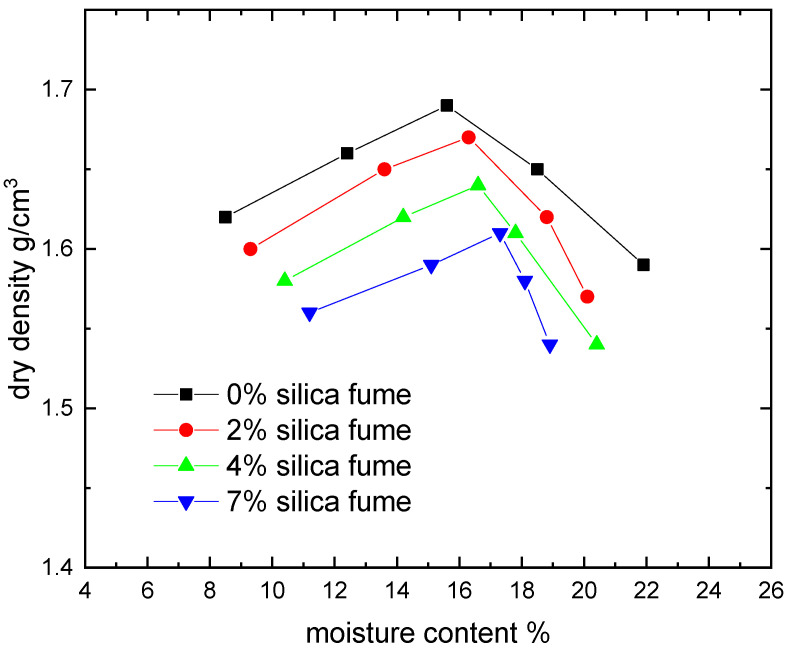
Effect of silica fume content on moisture content-dry density relationships.

**Figure 7:**
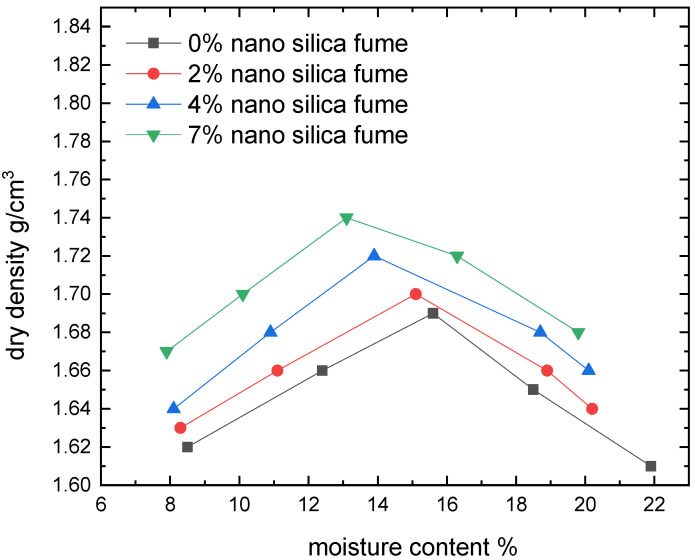
Effect of nano silica content on moisture content-dry density relationships.

**Figure 8:**
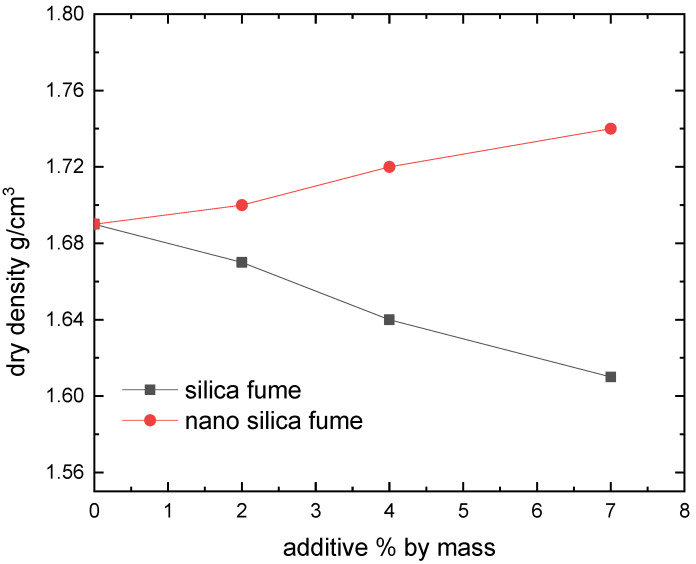
Dry density response to the change in additive.

**Figure 9:**
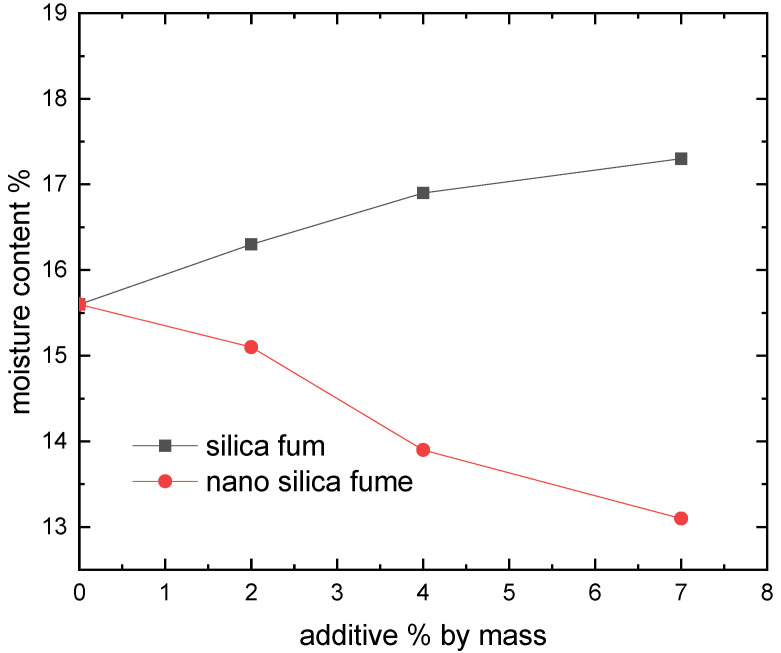
Effect of adding material on the optimum moisture content.

Figure 6, Figure 7, Figure 8 and Figure 9 show the compaction behavior of soil-silica fume and soil-nano silica mixes. These numbers came from five samples that were examined for each stabilizer percentage. According to these data, silica fume addition lowers maximum dry density and raises optimal moisture content, whereas nano-silica addition increases maximum dry density and decreases optimal moisture content for the studied samples. The substitution of soil with lower specific gravity for soil with a higher specific gravity led to a lowered maximum dry density when silica fume was added. In addition, silica fume gives samples a greater surface area than raw soil does. This suggests that additional water is required to compact the mixes before the ideal moisture content is raised. Because silica fume acts as a drying agent and adding micro material may increase the optimal moisture level by causing the compound to absorb more water due to pozzolanic reactions, more water is required to compact the soil-compound mixes. Additionally, ordinary materials are regarded as coarse materials in comparison to nanomaterials; therefore, when such additives interact with soil, the treated soil tends to be a coarser material with large surface areas formed; as a result, these processes require additional water to be carried out and decrease the amount of free clay fractions [15].

The tendency of nanomaterials to absorb water from moist soil, which decreases the ideal water content in soil due to the high surface area of nanomaterial particles, maybe the reason for the decrease in the optimal moisture content in soil treated with nanomaterials [27]. Figure 8 shows that adding micromaterials causes a reduction in maximum dry density, whereas adding nano-materials causes an increase. The coating of the soil by the compound mixing, which results in large particles with large voids (increasing particle size leads to an increase in void ratio) and therefore reduced density, may be attributed to the cause beyond the reduction in the case of adding silica fume [15].

#### 2.5.3. Unconfined Compressive Strength

**Figure 10:**
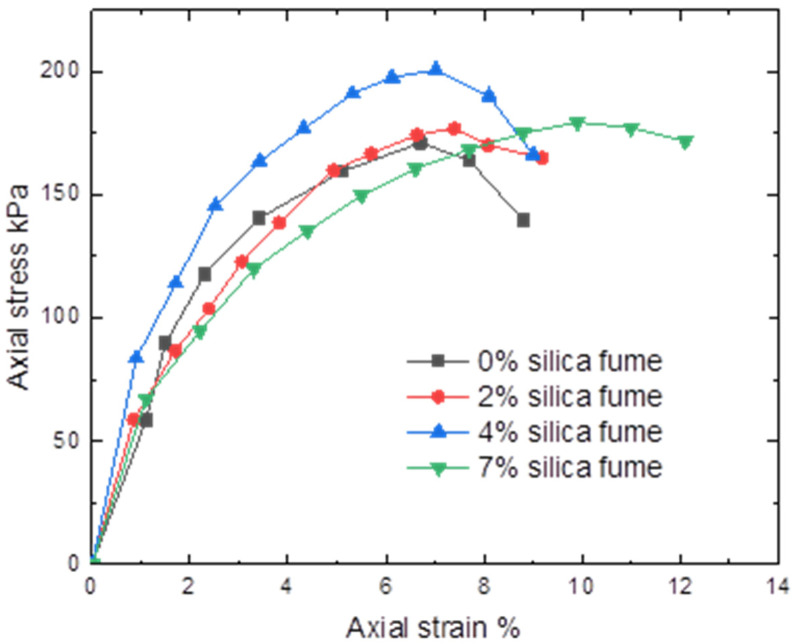
Axial stress versus strain for used soil with different percentages of silica fume.

**Figure 11:**
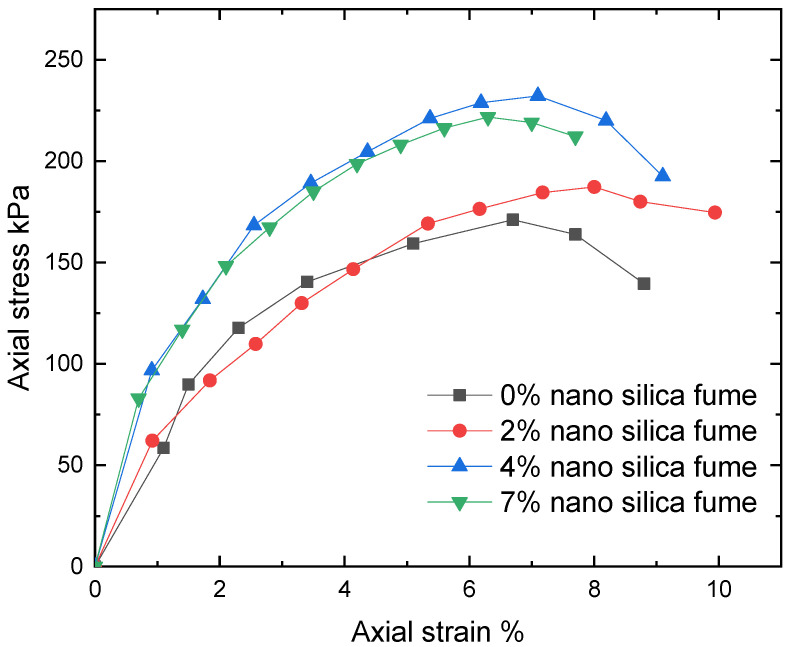
Axial stress versus strain for used soil with different percentages of nano silica.

Studies were carried out to investigate the impact of silica fume and nano-silica on the clay from Hujaira’s unconfined compressive strength. Figure 10 and Figure 11 show the average of these measurements for three replicates of each sample.

It may be determined that the unconfined compressive strength increased with the addition of silica fume and nano-silica. This is linked to the pozzolanic reaction between silica fume and clayey soil, which produces cementitious materials compounds that bind soil aggregates and may be caused by internal friction of silica fume particles. The unconfined compression test is frequently employed as a quick and affordable way to determine the cohesive soil’s approximative compressive strength. It is important to note that adding 4% silica fume as nano and micro materials results in the tested samples having the maximum strength.

## 3. Conclusions

The current study looked into how silica fume as nano and micro materials affected the compaction characteristics and strength of clayey soil known locally as Hujaira’s clay. The results demonstrate that silica fume in the form of nanomaterial can be used to improve the compaction characteristics and strength of poor strength clayey soil, known locally as Hujairas clay. Differently from the micro silica, which has a positive impact on the unconfined compressive strength, not the compaction characteristics. The following conclusions may be drawn from the current research:

- The Atterberg limits test results show that the addition of silica fume decreases the plasticity index of the treated soil while the latter increases with the addition of nano silica fume.
- Silica fume addition lowers the maximum dry density and increases optimum moisture content, whereas nano-silica addition increases maximum dry density and decreases optimal moisture content for the studied samples.
- The addition of 4% silica fume or nano silica lead to increasing the unconfined compressive strength of the tested samples.

## Figures and Tables

**Table 1:** Properties of clayey soil used in the present research.

Property	Clayey Soil
Unconfined compressive strength (kPa)	171
Liquid Limit LL (%)	52.6
Plastic limit (%)	35.3
Plasticity Index (%)	17.3
Specific Gravity, GS	2.72
Sand Content (%)	0
Silt Content (%)	37
Clay Content (%)	63
Max. Dry density (g/cm^3^)	1.69
Optimum Moisture Content (%)	15.6

**Table 2:** Chemical composition of the presently used silica fume.

Property	Composition (%)
SiO_2_	94.3
Al_2_O_3_	0.31
Fe_2_O_3_	0.82
SO_3_	0.91
C_a_O	0.29
M_g_O	0.133
K_2_O	0.442
Na_2_O	0.081
Tio_2_	<0.02
Loss on ignition	3.38

**Table 3:** Chemical requirement of pozzolans ASTM C618.

Oxide Composition	Pozzolan Class N
SiO_2_ + Al_2_O_3_ + Fe_2_O_3_ (min. percent)	70
SO_3_ (max. percent)	4
Moisture content (max. percent)	3
Loss on ignition max.	10

## Data Availability

Data is available from the corresponding author upon request.
